# Comment on “Digital Mental Health and COVID-19: Using Technology Today to Accelerate the Curve on Access and Quality Tomorrow”: A UK Perspective

**DOI:** 10.2196/19547

**Published:** 2020-04-27

**Authors:** Pauline Whelan, Charlotte Stockton-Powdrell, Jenni Jardine, John Sainsbury

**Affiliations:** 1 Centre for Health Informatics University of Manchester Manchester United Kingdom; 2 CAMHS.Digital Research Unit Greater Manchester Mental Health NHS Foundation Trust Manchester United Kingdom; 3 Greater Manchester Mental Health NHS Foundation Trust Manchester United Kingdom

**Keywords:** digital mental health, digital psychiatry, COVID-19, mhealth, mobile apps, learning health system

This letter is in response to the article published by Torous et al [1], which highlighted the potential of digital mental health in improving the accessibility and quality of mental health service provision during and beyond the coronavirus disease (COVID-19) pandemic. We write from a UK perspective to add to the North American lens of Torous et al. We contribute our personal and multidisciplinary insights as members of a digital health software team at a UK research University (PW and CS-P), an innovation manager of a mental healthcare provider in England (JS), a digital mental health research unit for young people (PW and JJ), and directors of a digital mental health Community Interest Company (PW and CS-P).

We agree that robust evaluation of mental health apps is important, particularly when selecting an appropriate app during emergency conditions. In the United Kingdom, a national Apps Library provides a central point for accessing trusted apps. In response to the COVID-19 pandemic, a dedicated list of useful apps has been established by ORCHA (Organisation for the Review of Care and Health Applications), an organization that advises the National Health Service on the safety and efficacy of health apps. Apps recommended on the national Apps Library and by ORCHA have been through an independent review and evaluation process and can guide health professionals and patients to trusted apps to support mental health problems including anxiety and depression. The evaluation process covers data security, clinical evidence, and user experience. More broadly, the NICE (National Institute for Health and Care Excellence) recommends that digital health technologies define a set of national UK evidence standards to guide development and evaluation of digital mental health systems [2].

Informal feedback from clinicians implementing rapidly introduced digital innovations within our mental health services has emphasized the need for ongoing evaluation. We see value in creating digital learning health systems [3] to support iterative quality improvement and have aimed to do this across digital innovation projects. Situating planned changes within the NASSS (Nonadoption, Abandonment, and Challenges to the Scale-Up, Spread, and Sustainability) framework [4] has helped us assess how digital health innovations can be safely and sustainably embedded in care pathways. We value digital training for mental health professionals, a view reflected in the recent UK Topol review consultation [5].

Like Torous et al, we are concerned about how digital technologies may exacerbate health inequalities. However, our experience during COVID-19 of moving a face-to-face young people’s digital mental health research group to an online videoconference has highlighted how digital solutions can overcome pre-existing (or previously invisible) barriers to participation. Our online group meetings make the group more accessible for certain members due to reduced travel, time required, personal preferences, or specific mental health conditions, which had made face-to-face group time difficult. Similarly, when face-to-face contact is impossible for physical distancing reasons during COVID-19, digital solutions that can provide remote support fill a critical gap. We believe that a more equitable distribution of digital resources and adequate digital literacy provision will promote a healthier digital experience for all.

## Abbreviations

COVID-19: coronavirus disease
NASSS: Nonadoption, Abandonment, and Challenges to the Scale-Up, Spread, and Sustainability
NICE: National Institute for Health and Care Excellence
ORCHA: Organisation for the Review of Care and Health Applications
